# Influenza Pandemic Waves under Various Mitigation Strategies with 2009 H1N1 as a Case Study

**DOI:** 10.1371/journal.pone.0014307

**Published:** 2010-12-20

**Authors:** Suma Ghosh, Jane Heffernan

**Affiliations:** 1 Department of Mathematics and Statistics, York University, Toronto, Ontario, Canada; 2 Center for Disease Modelling, York University, Toronto, Ontario, Canada; Yale University, United States of America

## Abstract

A significant feature of influenza pandemics is multiple waves of morbidity and mortality over a few months or years. The size of these successive waves depends on intervention strategies including antivirals and vaccination, as well as the effects of immunity gained from previous infection. However, the global vaccine manufacturing capacity is limited. Also, antiviral stockpiles are costly and thus, are limited to very few countries. The combined effect of antivirals and vaccination in successive waves of a pandemic has not been quantified. The effect of acquired immunity from vaccination and previous infection has also not been characterized. In times of a pandemic threat countries must consider the effects of a limited vaccine, limited antiviral use and the effects of prior immunity so as to adopt a pandemic strategy that will best aid the population. We developed a mathematical model describing the first and second waves of an influenza pandemic including drug therapy, vaccination and acquired immunity. The first wave model includes the use of antiviral drugs under different treatment profiles. In the second wave model the effects of antivirals, vaccination and immunity gained from the first wave are considered. The models are used to characterize the severity of infection in a population under different drug therapy and vaccination strategies, as well as school closure, so that public health policies regarding future influenza pandemics are better informed.

## Introduction

Influenza pandemics have been known to cause multiple waves of morbidity and mortality over a few months or years [1]. The cause of the wave behaviour of influenza pandemics is not precisely understood [2], [3]. Control measures such as vaccination and antiviral drugs will have an effect [4], [5], but to what extent do these need to be used to protect a population from severe infection? In June 2009, the World Health Organization declared the new strain of swine-origin H1N1 as a pandemic. Several countries combined antivirals and vaccinaiton strategies to battle the first and second waves of this pandemic. It is unknown, however, how effective these interventions have been on decreasing infection. School closure for the summer term in many countires may also have had an affect on disease spread. In this paper we provide estimates on the efficacy of antivirals and vaccination in the first and second waves of a pandemic, including a scenario of school closure in the summer months.

Vaccination is used to induce immunity in individuals such that, if they are exposed to the virus they have a high probability of resisting infection. Vaccination can also benefit a population by inducing herd immunity, where individuals that are not vaccinated are still protected from infection. Vaccination is the mainstay of seasonal influenza, however, in a pandemic situation the strain is initially unknown and the vaccine can take several months to be formulated. Thus, it is unlikely to be implemented in the first wave of infection, and may be available early in the second wave. However, the global manufacturing vaccine capacity is limited and is unlikely to meet the full demand of a pandemic threat. Also, the vaccine is developed from an early pandemic strain and if the strain changes over time, because of the high mutation rate of influenza, the vaccine will be less effective and only induce partial immunity.

Since efficacious vaccines are unlikely to be widely available during at least the first wave of pandemic influenza, antivirals, which reduce the ability of the virus to replicate but not provide immunity to a host, form a critical component for the containment of a pandemic. Antivirals may aid in the prevention of infection, but also reduce the severity of infection and the level of transmission [5]–[8]. Potential roles for antivirals include post-exposure prophylaxis (when drugs are given to individuals shortly after they are exposed), pre-exposure prophylaxis (when drugs are given before exposure) and early treatment (when drugs are given shortly after symptoms are presented).

During the 2009 H1N1 pandemic, vaccination and antivirals were employed to fight infection. Antivirals stockpiles of oseltamivir (Tamiflu) and zanamivir (Relenza), which were accumulated by many different countries in wait of the next pandemic threat, were used to provide prophylaxis and treat infections. In the beginning of the second wave, vaccination was also available. The use of antivirals and the rate of vaccine uptake, however, varied greatly by country (see Table 1). But, how do different control strategies affect the waves of morbidity and mortality of a pandemic?

**Table 1:** Antiviral stockpile size and number of doses of vaccine by country.

Country	stockpile	# doses	vaccine
	Size	vaccine	uptake
	(% population)	(million)	(% population)
Australia	41	21	30
Canada	25	50.4	40
China	1	100	3.2
France	50	94	7.8
UK	80	60	7
USA	30	195	20

In the 2009 H1N1 pandemic schools closed over the summer months. It has been shown that this can have substantial impact on the spread of an infectious disease which is transmitted through close contacts [9], [10]. Along with the use of antivirals, school closure must have had a great effect on disease spread in the first wave. But, does school closure change the most effective control strategy against a pandemic?

Mathematical modelling provides a toolkit that can be used to evaluate different control strategies of antiviral use and vaccine uptake, as well as school closure. Mathematical models have been employed to measure the efficacy of mitigation strategies of pandemic influenza considering pharmaceutical as well as non-pharmaceutical interventions [11]–[16]. They also rationalized the use of antiviral agents for both treatment and prophylaxis as a primary control measures during early stages of a pandemic [11], [12]. Since the start of the H1N1 pandemic a number of mathematical epidemiological studies have been used to understand the pandemic potential of the novel H1N1 strain early on [17], the initial transmission characteristics [18]–[20], and the disease burden and societal costs associated with infection [21]. Recently, mathematical studies have been used to evaluate the effects of a late release of a vaccine and closing schools [9], [22], [23] and the prophylactic use of antivirals [24].

A drawback of previous models of pandemic influenza is that they either ignore or do not explicitly consider the effects of immunity acquired from the first wave on disease outcomes in the second and consecutive waves. The underlying immunity of individuals can have a profound impact on the prevalence of disease in a population and the level of disease that is observed. This is seen through the correlation of transmissibility and immunity which are interlinked with the degree of susceptibility and disease outcome [25]. We have developed a mathematical model describing the first and second waves of an influenza pandemic, including antivirals, vaccination and summer school closure, that explicitly considers the effects of acquired immunity from the first wave of infection. This explicit consideration will aid in assessing pandemic control policies in a more informed manner.

## Methods

The mathematical model is composed of two smaller mathematical models describing the first and the second waves respectively. In both models we consider the effects of antivirals and/or vaccination that are available during that period. In the first wave we also consider the effects of summer school closures, and in the both waves we consider the effects of acquired immunity from previous infection either of the pandemic strain, or a seasonal influenza strain that may be closely related [26].

### First wave

#### Assumptions and Initial Conditions

The mathematical model of the first wave is an extension of an SEIR (susceptible-exposed-infected-recovered) model that includes the use of antivirals and the probability of asymptomatic infection.

Acquired immunity from previous infections of influenza that are related to the current circulating pandemic strain may have an effect on the size of the first wave through the effects of partial immunity [26]. It is, however, difficult to determine what percentage of the population, if any, has been infected by a strain that is related to the pandemic strain. It is also difficult to determine whether these individuals still maintain any immunity acquired from this previous infection. Since partial immunity may aid in preventing disease (symptoms) we include an asymptomatic class (A) in the model.

Antivirals form a critical component for the containment of a pandemic in the first wave. Antivirals may reduce the ability of the virus to replicate in a host and thus, will affect the level of virus transmission. It has been shown that to achieve effectiveness of antiviral treatment, therapy should be initiated within 48 hours of the onset of clinical symptoms [11], [12]. This is referred to as the window of opportunity (WOP) [12], [27]. It has also been shown that a delay between the onset of symptoms and the initiation of therapy can greatly affect the efficacy of treatment [28]. Early initiation of treatment appears to be the most important determinant of treatment efficacy [29]. Treatment started within the first 12 hours after the onset of fever can shorten the period of illness by more than 3 days as compared with treatment started at 48 hours [29] and treatment in later stages of the WOP can shorten the length of illness proportionately [29]. Since early administration of drug decreases the length of illness, we include two treated classes in the model: early the WOP, late in the WOP, and after the WOP. This model is similar to a previously published model by [28], but this model did not divide the WOP into two stages and thus, did not capture the effects of early versus late treatment.

We assume that viral transmission depends on the level of treatment and the degree of symptoms demonstrated. We thus, reduce the infectivity of the infected classes (asymptomatic, symptomatic with no treatment, symptomatic and treated in the first stage of the WOP, symptomatic and treated in the second stage of the WOP) proportionately. We also assume that immunity acquired from infection depends on the infected class. Thus, we include four recovered classes which correspond to each infected class. The resulting susceptible and recovered classes will be used as the initial population for the second wave model.

The first wave model is used to explore two scenarios of school closure. In the first scenario we assume that the first wave does not coincide with summer school closure and thus, model the first wave until the number of infections reaches zero (when the number of susceptibles is depleted). In the second scenario we consider the effects of summer school closure on the first wave. This is done by reducing the transmission parameter to correspond to a lower value of 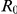 (the basic reproduction ratio) of the pandemic strain (see below).

#### First Wave Model

The first wave model considers a population comprised of individuals that are susceptible (S), exposed (E), asymptomatic infectious (A), untreated symptomatic infectious (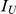), early treated symptomatic infectious (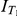), late treated symptomatic infectious (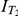), recovered from asymptomatic infection (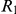), recovered from untreated symptomatic infection (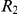), recovered from early treated symptomatic infection (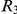) and recovered from late treated symptomatic infection (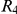). The model is as follows:
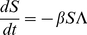
(1)

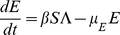
(2)

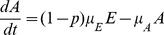
(3)

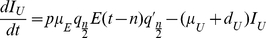
(4)

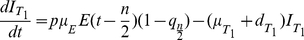
(5)

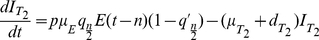
(6)

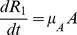
(7)

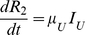
(8)

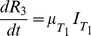
(9)

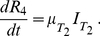
(10)


Here,
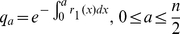
(11)

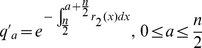
(12)represent the probability of infected individuals who remain untreated until age 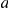 of clinical disease in the first and second stages of the WOP and depend on the rates of treatment in these stages, 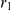 and 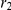 respectively. Also,
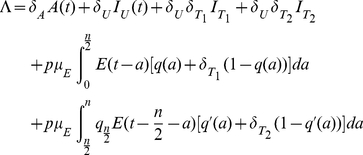
(13)is the force of infection, where 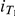, 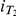, 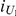 and 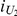 are the densities of treated and untreated individuals in the early and late stages of the WOP, given by
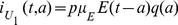
(14)

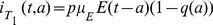
(15)for 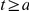 and
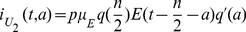
(16)

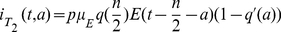
(17)for 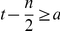.

**Figure 1:**
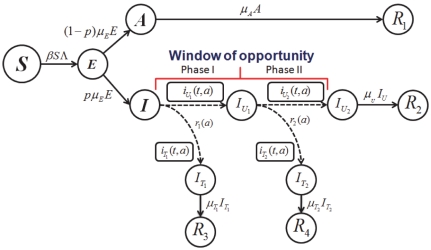
Schematic diagram of first wave model.

A schematic diagram for first wave model is depicted in Fig. 1. Model parameters and descriptions are listed in Table 2 and below. For the derivation of the model please see “Text S1”. Note that the recovered classes 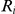
', 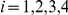 are distinguished because of different recovery rates from different infective classes and also because these classes will have different levels of acquired immunity which will affect the dynamics of the second wave.

**Table 2:** Population and parameters with description, values and sources for the 1st wave model.

Symbols	Description	Value	Source
Populations			
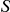	Susceptible		
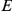	Exposed		
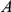	Asymptomatic		
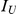	Symptomatic untreated		
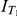	Symptomatic, treated day one of WOP		
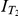	Symptomatic, treated day two of WOP		
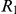	Recovered from 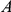		
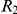	Recovered from 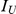		
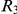	Recovered from 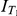		
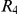	Recovered from 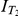		
Parameters			
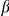	Baseline transmission rate of infection	variable	with school closure 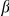 decreasing after 70 days
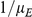	Mean incubation period	3 days	[21], [31]
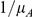	Mean infectious period of asymptomatic infection	4.1 days	[16]
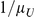	Mean infectious period of untreated symptomatic infection	2.85 days	[11], [28]
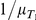	Mean infectious period of symptomatic if treated on day one WOP	1.05 days	[28], [42]
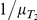	Mean infectious period of symptomatic if treated on day two WOP	2 days	[28], [42]
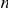	Length of the WOP	2 days	[12], [27]
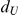	Death rate of untreated symptomatic infection	0.002/day	[28]
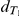	Death rate of symptomatic treated on day one of WOP	0.0001/day	[28]
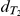	Death rate of symptomatic treated on day two of WOP	0.0002/day	[28]
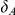	Relative infectiousness of asymptomatic infection	0.071	[28]
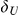	Relative infectiousness of untreated symptomatic infection	0.143	[28]
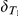	Relative infectiousness of treated (day one) symptomatic infection	0.3	[28] and assumption
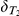	Relative infectiousness of treated (day two) symptomatic infection	0.4	[28]
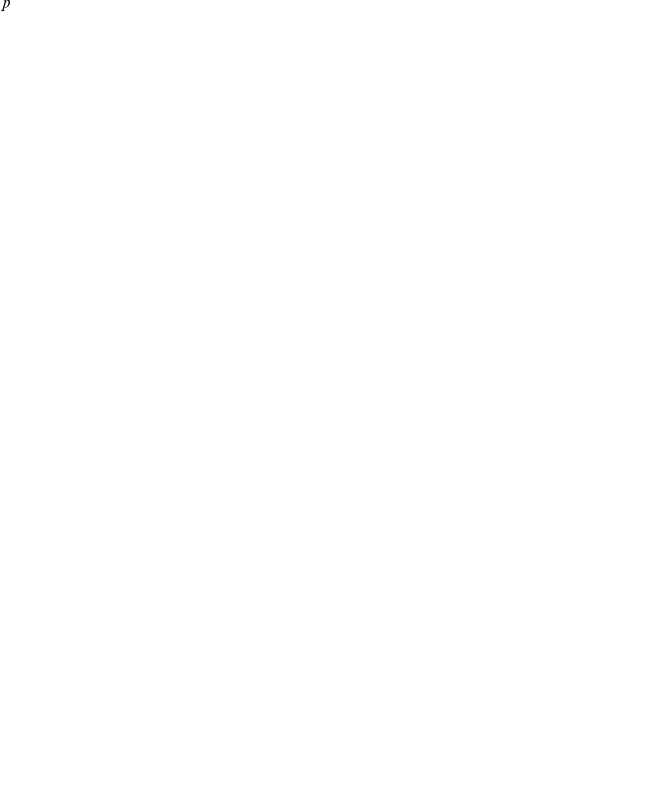	Probability of developing symptoms	0.6	[11], [16], [22], [24], [31]
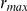	Maximum treatment level in WOP	0.4	

#### Reproduction numbers

The basic reproduction number (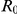), defined as the number of new cases caused by one infectious person entering a totally susceptible population (in the absence of any interventions) [30], is the key parameter used to determine whether an infection will spread in a population. For the first wave model, we find that
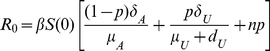
(18)where S(0) is the initial population of susceptibles. The three terms correspond to the contributions of asymptomatic infectious individuals and symptomatic infectious individuals during and after the WOP.

The control reproduction number 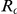 is another useful quantity that can be used to evaluate whether control measures or interventions can contain or halt pathogen spread. For the first wave model
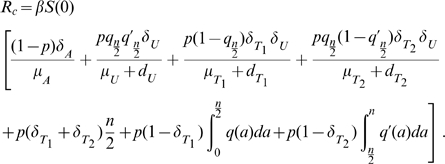
(19)


Note that, in absence of control measures (i.e. 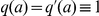) 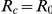.

#### Parameter Values

Parameter values for the first wave model are listed in Table 2. Briefly, we assume that 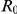 is near or in the reported range of 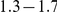 for the H1N1 pandemic [17]–[20]. When considering the effects of summer school closures we decrease the transmission paramter to reflect a lower 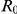, but keep it such that 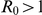. This was done in similar fashion to [9], [10]. We assume that after a short incubation period of 3 days [21], [31] 60% of infected individuals develop clinical symptoms [11], [16], [22], [24], [31] and treatment may commence at this time. The mean infectious period of symptomatic individuals who remain untreated is taken to be 4.85 days [28], [29] which includes the 2 day WOP [28], [29] and a mean duration of 2.85 days during which initiating treatment is not effective. The mean duration of asymptomatic infection is assumed to be 4.1 days [16] and antiviral treatment is assumed to reduce infectiousness by 60% from the time when treatment is initiated [11], [32]. Asymptomatic infection is assumed to be 50% less infectious than symptomatic cases [16]. The baseline transmission rate is calculated using a final size relation (see Text S1). The death rates of the symptomatic untreated and treated classes are taken from [28].

**Figure 2:**
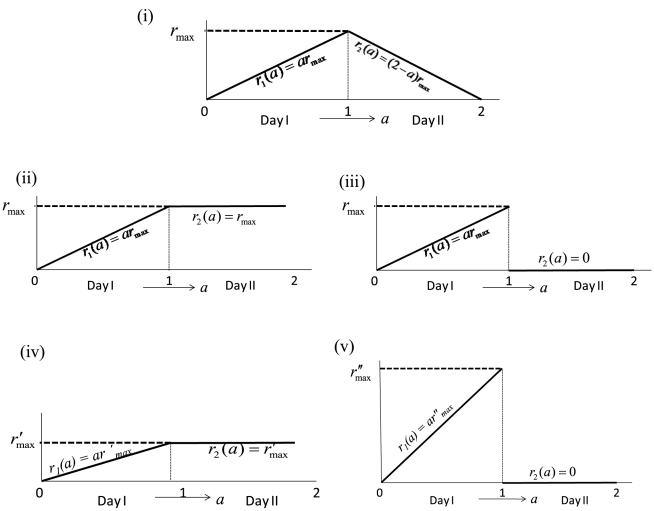
Profile of treatment rate - function (i–v). 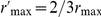
 and 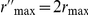.

To investigate the feasibility of containing a pandemic in the first wave with antivirals, we prescribe five different scenarios for the treatment rate (see Fig. 2). We assume that treatment may commence with the onset of symptoms and it can be administered for 2 days (the length of the WOP). We also assume that there is a maximum treatment level 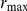. Treatment profiles (i–iii) were chosen to reflect the fact that antiviral stockpiles may be more limited in some countries over others. In these three profiles the treatment rate increases with slope 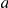 on the first day to the maximum level 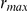. This is then followed by either (i) a decline with slope 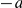 on the second day (Fig. 2(a)), (ii) a constant level at 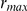 on the second day (Fig. 2(b)) or (iii) a constant level of zero (Fig. 2(c)). Treatment profile (i) reflects a situation which gives priority to individuals that are diagnosed in the mid-stages of the WOP. Treatment profile (ii) reflects a situation in which a country may have a large stockpile and can administer doses to individuals in the second stage of the WOP. Treatment profile (iii) represents a scenario in which the stockpile is limited, thus, antivirals are only given to individuals that present to the doctors in the first stage of the WOP, which has a greater probability of reducing infection and transmission. Treatment profiles (iv–v) are similar to profiles (ii–iii), but have the same total treatment rate (area under the curve) as profile (i) (see Fig. 2(d,e)). Profiles (iv–v) may be chosen to replace profile (i) if they are more effective in reducing infection and, perhaps, treat less infections. Treatment profiles (i–v) can be represented by:
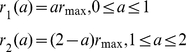
(20)

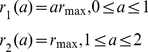
(21)

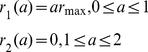
(22)

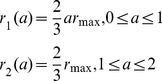
(23)

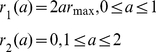
(24)


We assume that the treatment of clinical cases commences only after the first 30 days of the first wave. This reflects the fact that there is usually a lag between the incidence of disease in a population and clinical recognition of disease cases.

### Second wave

#### Assumptions and Initial Conditions

Immunity gained from infection in the first wave or vaccination will affect the severity of the second wave. Firstly, it will change the overall susceptibility of infection of the population, and secondly, it will affect the number of cases that develop symptomatic infection. We have developed a model of influenza transmission dynamics in the second wave that includes susceptible classes that are delineated by immune status from the first wave and by vaccination (see Table 3). It is assumed that the susceptibility of each class with some existing immunity will be reduced by factor 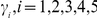, (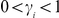) where 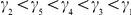.

**Table 3:** Population and parameters with description, values and sources for the 2nd wave model.

Symbols	Description	Value	Source
Populations			
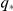	Not infected in the first wave		
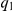	Recovered from asymptomatic infection in first wave		
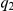	Recovered from symptomatic untreated infection in first wave		
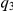	Recovered from treated symptomatic infection on day one of WOP in first wave		
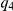	Recovered from treated symptomatic infection on day two of WOP in first wave		
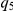	Vaccinated at beginning of second wave		
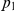	Asymptomatic infected in second wave		
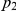	Symptomatic infected and untreated in second wave		
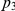	Symptomatic infected and treated on day one of WOP in second wave		
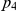	Symptomatic infected and treated on day two of WOP in second wave		
Parameters			
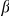	transmission rate in the second wave	0.9302 and 1.0148	when no school closure in the first wave
		0.6342	when school closure is considered in the first wave
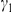	Reduction in susceptibility of 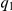	0.75	Assumption
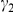	Reduction in susceptibility of 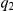	0.25	Assumption
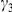	Reduction in susceptibility of 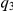	0.5	Assumption
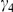	Reduction in susceptibility of 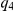	0.4	Assumption
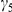	Reduction in susceptibility of 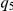	0.3	Assumption
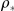	Probability of symptomatic infection of 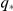	0.6	Assumption
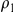	Probability of symptomatic infection of 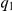	0.5	Assumption
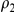	Probability of symptomatic infection of 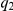	0.2	Assumption
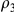	Probability of symptomatic infection of 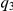	0.4	Assumption
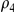	Probability of symptomatic infection of 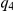	0.3	Assumption
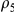	Probability of symptomatic infection of 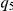	0.25	Assumption
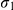	Reduction in infectiousness of 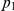	0.2	Assumption
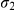	Reduction in infectiousness of 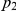	0.7	Assumption
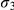	Reduction in infectiousness of 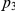	0.3	Assumption
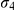	Reduction in infectiousness of 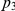	0.4	Assumption
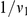	Mean infectious period of 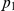	4.1 days	[16]
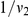	Mean infectious period of 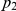	4.85 days	[11], [28]
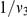	Mean infectious period of 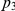	3.05 days	
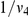	Mean infectious period of 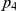	4 days	
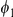	Probability of treatment on day one of WOP	Variable	
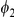	Probability of treatment on day two of WOP	Variable	

We include infected classes that are delineated by immune status (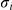, 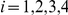), (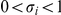) at the time of exposure (see Table 3). It is assumed that the development of symptomatic infection is intimately linked to the immune status of the individual at the time of exposure to the pathogen [25]. Thus, we assume that the probability of asymptomatic infection increases by strength of pre-existing immunity at the time of exposure. We take 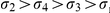.

#### Second wave model

The effect of partial immunity on seasonal influenza epidemics has been studied through history-based formulations [33]–[35] and status-based models [36], [37]. We propose a second wave model that is related to these models in that it includes reduced susceptibility of a susceptible host population and reduced transmissibility of infected individuals. The model is an extension of the classical SIR model [38] and can be written as
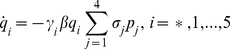
(25)

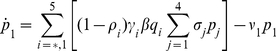
(26)

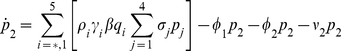
(27)

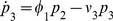
(28)

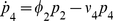
(29)

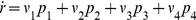
(30)where 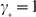, 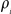 is the probability of symptomatic infection, 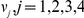 are the recovery rates of respective classes of infected individuals and 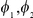 are the proportion of symptomatic infected individuals initiating treatment on the first and second stages of the WOP respectively.

**Figure 3:**
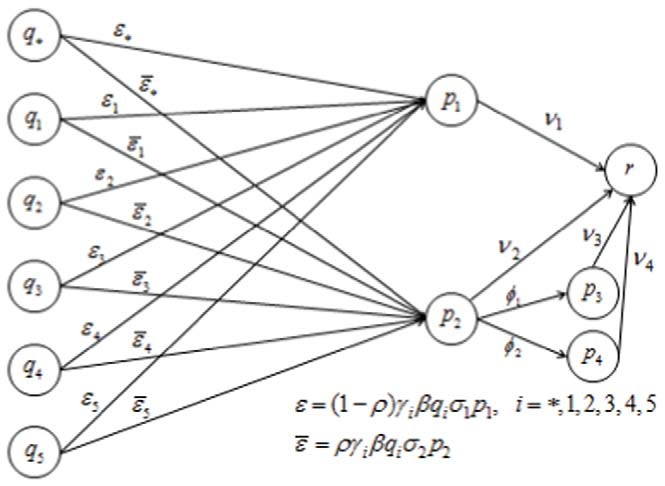
Schematic diagram of second wave model.

If 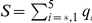, 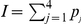 and 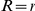 and 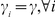, 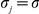 and 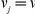, 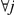 then the above model reduces to the SIR model
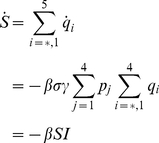
(31)

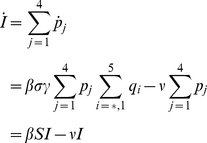
(32)

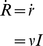
(33)where 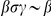. A schematic of the second wave model is shown in Fig. 3. Parameters and descriptions are listed in Table 3 and below. Note that the we chose not to employ a similar model to Eq. 10 for the second wave since vaccination would dominate the infection outcomes and, thus the effects of the different treatment profiles in the second wave would be negligible.

#### Reproduction numbers

The number of secondary cases in the second wave is given by the control reproduction number
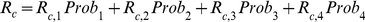
(34)where
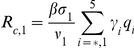


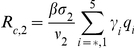


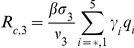


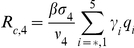
which give the number of secondary infections produced by an infected individuals that is asymptomatic, symptomatic and untreated, symptomatic and treated on the first stage of the WOP and symptomatic and treated on the second stage of the WOP, and
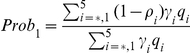


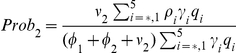


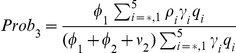


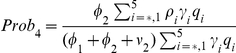
are the probabilities of infection being initiated by an individual that is asymptomatic, symptomatic and untreated, symptomatic and treated on the first stage of the WOP or symptomatic and treated on the second stage of the WOP.

In the absence of vaccination and antiviral treatment in the second wave 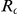 reduces to the effective reproduction number
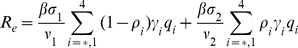
(35)and if no prior immunity against the pathogen exists it further reduces to the basic reproduction number
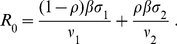
(36)


#### Parameter values

Parameter values are similar to those used in the first wave and were taken from the modelling and clinical literature of influenza A and H1N1. When we do not consider the effects of summer school closure we assume that 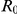 in the second wave is greater than 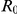 for the first wave so that infection of the population with partial immunity will still occur (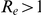). This reflects the fact that in some pandemic situations the second wave may be started by an imported case of a mutated and higher fit strain than what was present during the first wave of a pandemic. Here, we have chosen to study 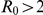 such that 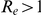. When summer school closure is considered, we increase the transmission rate back up to the value used in the beginning of the first wave when schools are openned for the next school term. This reflects the fact that social contacts are increased when school returns. We also chose values for reduced susceptibility and symptomatic infection so that the relationships 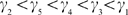 and 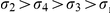 are satisfied.

The initial population of the second wave will depend on the treatment profile of the first wave (see Results). It is assumed that the same proportion of the resulting susceptible and recovered classes from the first wave are vaccinated.

## Results

We consider two scenarios of school closure. We first consider the case when the pandemic occurs at a time when summer school closure will not coincide with the first or second wave. We then consider the case when summer school closure occurs during the first wave of infection similar to that experienced in the 2009 H1N1 pandemic. In both scenarios we first simulate the first wave model Eq. 10 to evaluate the impact of different treatment profiles on disease incidence. We then simulate both models, using the results of the first wave model to initialize the second wave model Eq. 30 to compare and contrast different combinations of treatment and vaccination strategies.

### No school closure in the first wave

#### First wave

**Figure 4:**
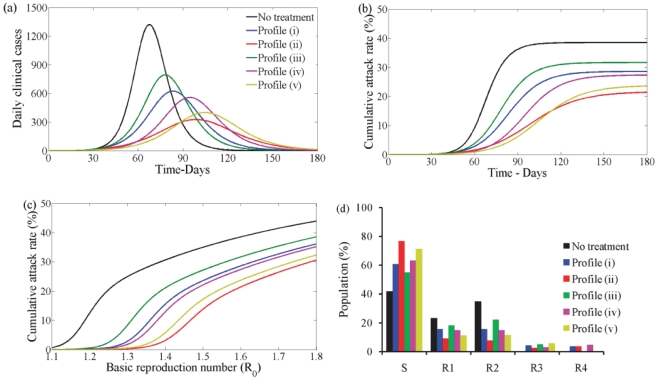
First wave with and without treatment with no school closure. (a) Disease incidence with no treatment (black line) and with treatment profiles (i–v) (blue, red, green, pink, yellow) when 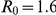. (b) Cumulative attack rate under no treatment and treatment with profiles (i–v) when 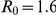. For each profile of treatment the cumulative value at the end point corresponds to the clinical attack rate given by 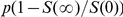, where 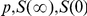 respectively gives the probability of getting symptoms, the final value of susceptible at the end of epidemic and the initial susceptible population in the beginning. (c) Cumulative number of infections with no treatment and treatment profiles (i–v) when 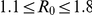. (d) Distribution of resulting susceptible and recovered classes from first wave for no treatment and treatment profiles (i–v).

Fig. 4(a,b) shows the progression of infection in a population and the cumulative attack rate (untreated and treated infections) over the first wave when 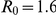 for each treatment profile ((i) - blue, (ii) - red, (iii) - green, (iv) - pink and (v) - yellow) and when treatment is not used (black). When treatment is not used, the first wave infection peaks at 1.3% of the population around day 60–70 and 38% of the population experiences infection over the whole wave. As the treatment rate increases the wave peak decreases in magnitude and occurs later in time Fig. 4(a). The cumulative attack rate also decreases Fig. 4(b).

As 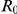 increases, the wave peak increases (not shown) and the proportion of the population experiencing infection also increases with and without treatment (Fig. 4(c)). When treatment is used, the cumulative attack rate is greatly reduced (Fig. 4(c)). When 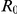 is only slightly greater than one the five treatment profiles result in a similar number of total infections (Fig. 4(c)). However, as 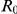 increases they diverge but follow similar dynamics: there will be a steep increase in the number of infections and this will be followed by an approximately linear relationship with 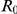 (Fig. 4(c)).

The distribution of the susceptible and recovered classes at the end of the first wave depends on the value of 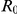 (not shown) and also on the treatment profile used (Fig. 4(d) assuming 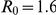). The proportion of the population receiving treatment is also affected i.e. if 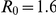, 7.7, 6.1, 4.9, 7.3 and 5.6% of the population received some form of treatment under treatment profiles (i–v) respectively (Fig. 4(d)). The resulting distribution will provide the starting population of the second wave.

Comparing the different treatment profiles we see that profile (ii) always results in the lowest amount of infection. The overall treatment efficacy however, may not be the best. When 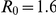 over 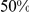 of those treated under treatment profile (ii) received drug therapy only in the second stage of the WOP (Fig. 4(c,d)) which is less effective at reducing disease and transmission. In contrast, treatment profile (v) which results in only a slightly higher level of infection (Fig. 4(b,c)) uses less drugs and all those treated were given treatment on the first day (Fig. 4(d)).

#### Second wave

It is not known what the basic reproductive ratio of the second wave of a pandemic will be and it cannot be measured since partial immunity in the population exists. It is possible that the second wave will be initiated by a more fit strain of H1N1 in that the transmission rate increases. It is also possible that the virus will have evolved so that individuals that were infected in the first wave are not fully immune. In this section we have chosen values for the transmission rate 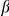 so that 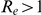 for the initial population resulting from any of the five treatment profiles in the first wave and when vaccination is used at the start of the second wave. It is assumed that, when vaccination is used, the same proportion of individuals from each susceptible class will be vaccinated. The 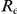 values are 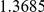 and 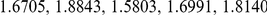 corresponding to no treatment and using treatment profile (i–v) respectively when 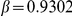 in the second wave. We also look at a second case for a more fit strain with 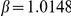, the respective values of 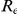 are 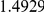 and 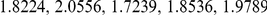.

In this section we have also chosen values for 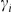 and 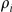, 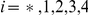 to reflect reduced levels of susceptibility and a reduced probability of symptomatic infection depending on the infection history in the first wave.

**Figure 5:**
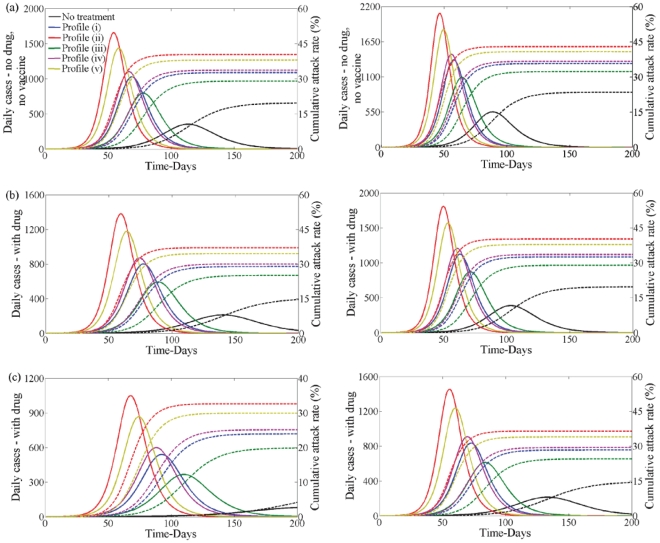
Disease incidence in second wave without vaccine and no school closure in first wave. Clinical infection in second wave with no vaccine when 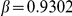 (left) and 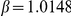 (right) having no school closure in the first wave. Lines correspond to no treatment (black) or treatment following profile (i–v) (blue, red, green, pink, yellow) in the first wave when 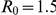. In each plot disease incidence and cumulative attack rates are shown. (a) No drug or vaccine. (b) With drug but no vaccine. Drug uptake is 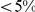. (c) With drug but no vaccine. Drug uptake is 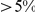.

Second wave without vaccination - Fig. 5(a) (black line) shows the case when drug therapy and vaccination are not used during the first and second waves when 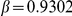 (left) and 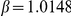 (right). Comparing the first and second waves (Fig. 4(a) and Fig. 5(a)) we see that the peak is decreased in the second wave and it occurs at a later time. The cumulative attack rate is also lower (Fig. 4(b) and Fig. 5(a)).

When drug therapy is used in the first wave, the second wave is more severe (Fig. 5(a) coloured lines) compared to when it is not (Fig. 5(a) black line). The wave peak is higher and occurs earlier in time. The cumulative attack rate is also greater. Thus, drug therapy interventions in the first wave have a substantial impact on the second wave. This is a direct effect from the difference in the underlying immunity of the susceptible population at the beginning of the second wave.

In Fig. 5(b,c) we show the prevalence of infection and cumulative attack rate in the second wave when 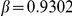 (left) and 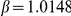 (right) when drug therapy is used to treat infected individuals in the second wave. Fig. 5(b) reflects the scenario when the antiviral stockpile is limited and Fig. 5(c) reflects the situation when a large antiviral stockpile exists. The prevalence of infection and the cumulative attack rate is reduced when drug therapy use increases. However, if 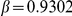 and drug uptake is 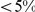 (Fig. 5(b), left) the magnitude of the second wave is similar to the case when 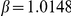 and drug uptake is 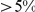 (Fig. 5(c), right).

Note that, if drug therapy is used in the first wave, profile (iii) always results in the lowest number of infections whether drug therapy is used in the second wave or not.

**Figure 6:**
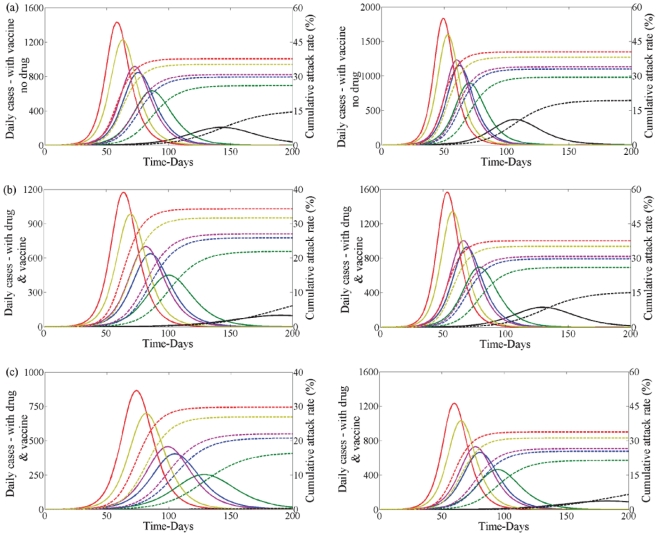
Disease incidence in second wave with vaccine and no school closure in first wave. Clinical infection in second wave with vaccine when 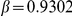 (left) and 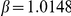 (right) having no school closure in the first wave. Lines correspond to no treatment (black) or treatment following profile (i–v) (blue, red, green, pink, yellow) in the first wave when 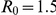. In each plot disease incidence and cumulative attack rates are shown. (a) With vaccine but no drug. (b) With drug and vaccine. Drug uptake is 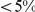. (c) With drug and vaccine. Drug uptake is 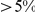.

Second wave with vaccination - Vaccination affects the severity of the second wave of infections. When only vaccination is used the peak of the second wave is reduced in magnitude and occurs later in time compared to the case when only drug therapy is used (Fig. 6(a) and Fig. 5(a)). In turn, it reduces the cumulative attack rate in the second wave (Fig. 6(a) and Fig. (5a)). When both vaccination and drug therapy is available in the second wave these are also further reduced (Fig. 6(b,c) and Fig. 5(b,c)). When 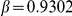 and drug uptake is 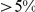 (Fig. 6(c) left) the second wave is almost non-existent for the case when drug therapy is not used in the first wave (black line). Note that, if drug therapy is used in the first wave, profile (iii) always results in the lowest number of infections whether drug therapy is used in the second wave or not.

**Figure 7:**
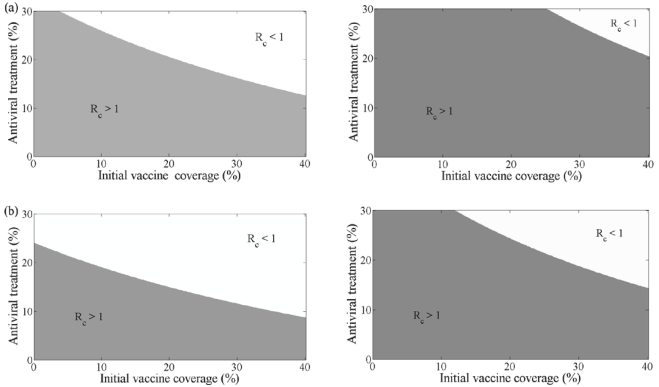
Combined effect of antiviral and vaccine during second wave with no school closure in first wave. The combined effect of antiviral treatment and initial vaccination in reducing the control reproduction number (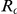) during the second wave of the pandemic with 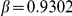 (left) and 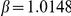 (right) having no school closure in the first wave. The white region shows the eradication of the disease where the grey region shows disease persistence. (a) Profile (i) used in first wave. (b) Profile (iii) used in first wave.

Fig. 7 plots the combined effect of antiviral treatment and initial vaccination in reducing the control reproduction number 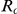 in the second wave for treatment profiles (i) and (iii) when 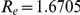 and 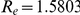 corresponding to 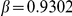 in the second wave. This figure shows that as vaccination uptake increases, the need for drug therapy to control infection in the second wave reduces. Also, when drug therapy use increases, the need for vaccination to control the second wave decreases. This is true for all treatment profiles used in the first wave, however, treatment profile (iii) results in a distribution of susceptibles of the second wave that is more sensitive to the effects of vaccination and drug therapy uptake in the second wave.

**Figure 8:**
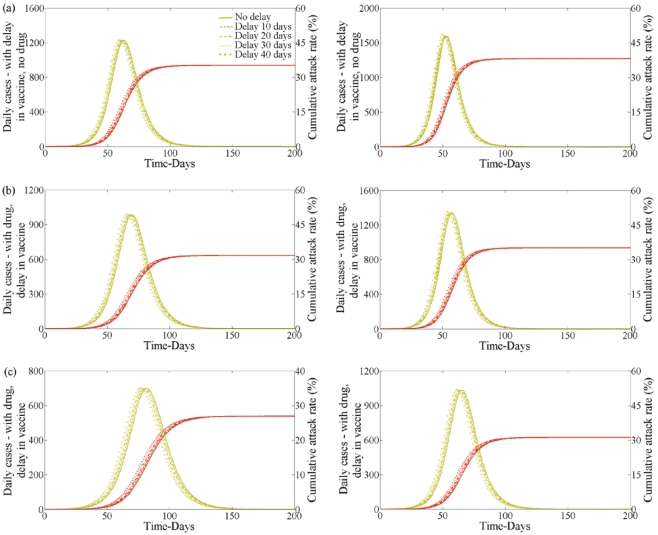
Delaying vaccination in second wave with no school closure in first wave. Effect of delay in initiating vaccination for second wave of clinical infection when 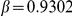 (left) and 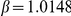 (right) having no school closure in the first wave. Lines correspond to 0, 10, 20, 30 and 40 days delay. Initial distribution results from profile (v) in the first wave when 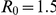. In each plot disease incidence and cumulative infections are shown. (a) With vaccine but no drug. (b) With drug and vaccine. Drug uptake is 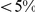. (c) With drug and vaccine. Drug uptake is 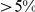.

Vaccination may not be available at the beginning of the second wave. A delay of up to 40 days in the release of the vaccine will have little to no effect on the second wave peak and on the cumulative attack rate (Fig. 8 for treatment profile (v) in the first wave). This is true for all treatment profiles (not shown).

#### Both waves

**Figure 9:**
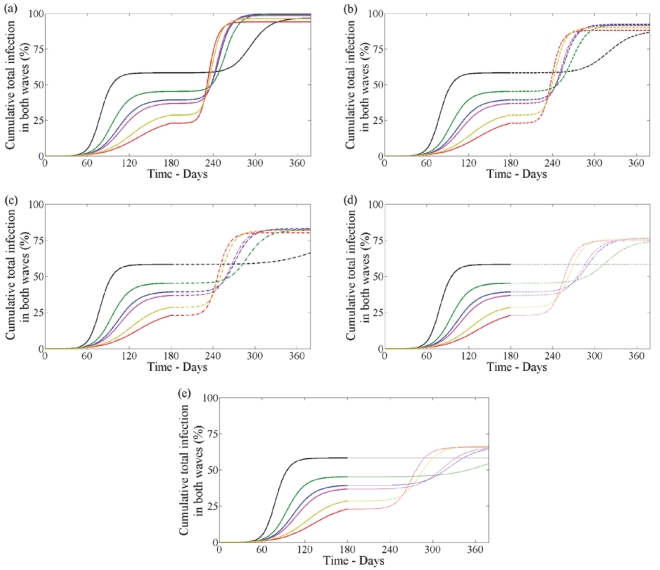
Cumulative total infections in both waves with no school closure in first wave. Cumulative infections (clinical and subclinical) in both waves when 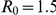 in the first wave having no school closure. The five panels correspond to second wave: (a) no drug or vaccine (solid line), (b) no vaccine with drug 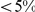 (dashed line), (c) no vaccine with drug 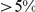 (dashdot line), (d) with drug uptake 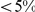 and vaccine coverage 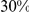 (dotted line), (e) with drug uptake 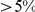 and vaccine coverage 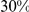 (solid line with circles). In each panel lines correspond to no treatment (black) and treatment profile (i–v) (blue, red, green, pink, yellow) in the first wave.

The goal of a pandemic control strategy is ultimately to reduce the total level of infection. Fig. 9 shows the cumulative number of infections (asymptomatic, symptomatic untreated and symptomatic treated on either day) over both waves for all treatment profiles in the first wave, and all combinations of drug therapy and vaccination in the second wave. This figure shows that the cumulative number of cases will lie between 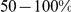 of the population depending on the control strategy in both waves.

Comparing all combinations of strategies in the first wave and second wave (Table 4(a) with 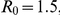 in the first wave and 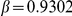 in the second wave), we find that a control strategy of vaccination and high use of drug therapy in the second wave will always result in the lowest number of cases no matter what treatment profile is used in the first wave. The lowest level of infection over both waves, 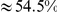 of the population, results from a combination of treatment profile (iii) in the first wave and vaccination and 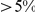 drug uptake in the second wave. However, if drug therapy uptake in the second wave is low or vaccination is not available in the second wave, then the best strategy to minimize the total number of infections over both waves is to use no drug therapy in the first wave. If both vaccination and drug therapy are not available in the second wave, profile (ii) in the first wave results in the lowest number of total infections.

**Table 4:** Cumulative (a) total infections and (b) clinical cases over both waves when 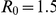 in first wave without school closure and 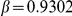 in the second wave.

			Second wave				
			no drug	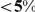	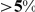	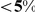	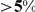
			no vaccine	no vaccine	no vaccine	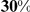	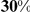
(a)	First wave	no treatment	96.7	86.7	66.2	58.4	58.3
		profile (i)	99.4	92.3	83.0	76.4	64.3
		profile (ii)	94.1	88.2	80.3	74.7	65.8
		profile (iii)	99.6	92.0	82.1	74.0	54.5
		profile (iv)	98.5	91.6	82.5	76.1	65.0
		profile (v)	96.3	90.0	81.7	75.9	66.5
(b)	First wave	no treatment	54.6	49.6	39.1	35.0	35.0
		profile (i)	56.2	52.5	47.5	43.2	36.9
		profile (ii)	54.1	50.9	46.5	42.3	37.5
		profile (iii)	56.2	52.3	47.0	42.1	32.0
		profile (iv)	55.8	52.1	47.2	42.9	37.2
		profile (v)	55.1	51.7	47.1	43.0	37.9

All infections are not visible to public health as some may be asymptomatic. Table 4(b) lists the cumulative number of clinical cases (treated and untreated, cumulative attack rate) over both waves assuming 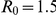 in the first wave and 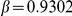 in the second wave. To reduce the total number of clinical cases treatment profile (iii) in the first wave along with vaccination and 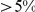 drug uptake in the second wave still results in the lowest number. Also, if drug uptake is low in the second wave, or if vaccination is not available in the second wave, then no treatment in the first wave still results in the lowest number, and profile (ii) in the first wave will result in the lowest number of clinical cases if there is no vaccination or drug available in the second wave.

If the vaccination level is lower than 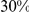 uptake in the second wave, then no treatment in the first wave may result in a lower number of infections than treatment profile (iii) when vaccination and drug therapy (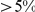) are available in the second wave (not shown). Also, profile (ii) may replace no treatment in the first wave as the best strategy if vaccination uptake is low when drug therapy uptake is 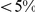 in the second wave (not shown). In both of these cases, however, the difference between no treatment and profile (iii), and no treatment and profile (ii) is very small (not shown).

### School closure in the first wave

School closure during the summer vacation may result in a reduction in transmissibility of H1N1 in the first wave [9]. The closing of schools over the summer months will reduce the number of contacts of school age children, thus affecting the transmissibility of the H1N1 pandemic virus which may cause a great decrease in infections. This could be seen as the first wave of the pandemic. In this section school holidays are assumed to start 70 days after the first wave emerges and last approximately 60 days (July and August). This gives a total duration of 130 days of first wave of the pandemic (starting from last week of April). The second wave is considered for 180 days (from September to February of the next year). As before, we simulate the first wave model to evaluate the different treatment profiles on disease burden and then we simulate both models using the resulting distribution of the susceptible and recovered populations at the end of the first wave.

#### First wave

**Figure 10:**
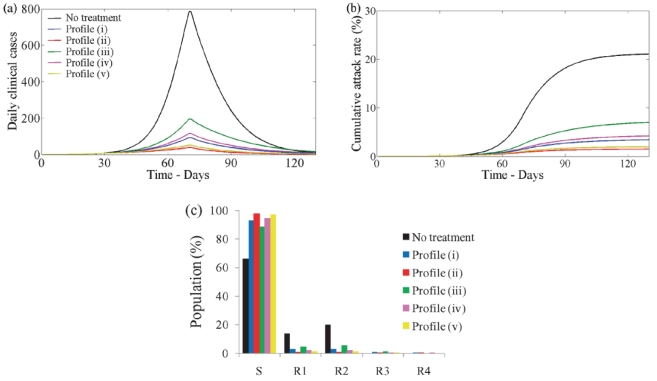
Disease incidence in first wave with and without treatment and school closure for last 60 days. First wave with and without treatment considering school closure in the first wave from the day 71 to day 130. (a) Disease incidence with no treatment (black line) and with treatment profiles (i–v) (blue, red, green, pink, yellow) when 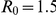. (b) Cumulative attack rate under no treatment and treatment with profiles (i–v) when 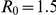. For each profile of treatment the cumulative value at the end point corresponds to the clinical attack rate. (c) Distribution of resulting susceptible and recovered classes from first wave for no treatment and treatment profiles (i–v).

As the contact pattern changes due to school closure approximately 70 days after the first wave emerges, we assume that the transmissibility of the virus reduces by 30% (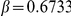) of the initial transmissibility (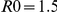 initially) [10]. Fig. 10(a) shows that if there is no treatment the first wave infection peaks at 0.8% of the population around day 70 and it comes down as the transmissibility reduces observably during the summer holidays. Approximately 21% of the population experiences infection over the whole wave without treatment (Fig. 10(b)). Treatment with the different profiles (i–v) reduces the wave peak in magnitude (Fig. 10(a)). The cumulative attack rate also decreases accordingly due to treatment in the school closure scenario (Fig. 10(b)). Note that total infection is greatly reduced in this scenario when compared to the case when school closure does not occur.

Fig. 10(c) shows the distribution of the susceptible and recovered populations for all scenarios of treatment in the first wave (i–v). This figures demonstrates that school closure over the summer months increases the naive susceptible population to levels close to 100% (93.1, 97.6, 88.6, 94.4 and 96.8% for profile (i–v) respectively) for second wave. For treatment profiles (i–v), 1.3, 0.6, 1.2, 1.2 and 0.6% of the population received some form of treatment respectively (Fig. 10(c)). The distribution of the susceptible and recovered populations from the first wave provides the starting population at the beginning of the second wave, when schools reopen for the next school year.

#### Second wave

In the 2009 H1N1 pandemic the first dip of infection (or first wave) was probably caused by the end of the school term [9]. However, when school returns the transmissibility which was reduced during summer vacation can then be restored to its original value in the second wave. In the second wave we find that the 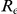 values are 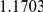 and 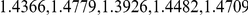 corresponding to no treatment and using treatment profile (i–v) where the minimum is with profile (iii) and the maximum is with profile (ii) when treatment is considered. As before, we assume that the same proportin of individuals from each susceptible class is vaccinated.

**Figure 11:**
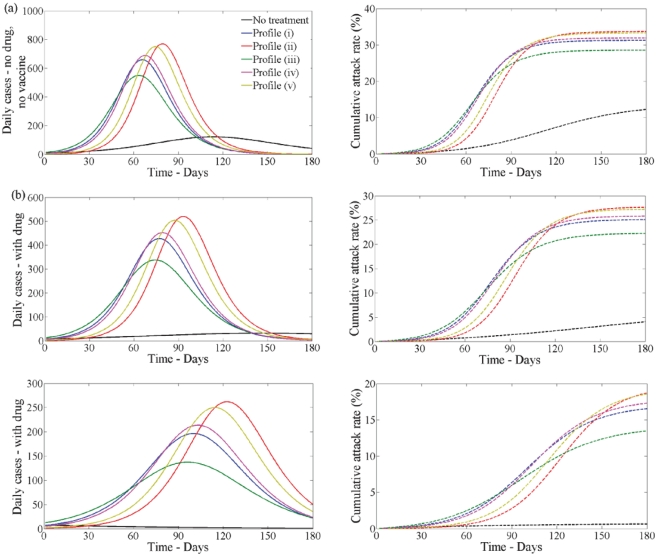
Disease incidence in second wave without vaccination and with school closure in first wave. Clinical infection in second wave with no vaccine (left) and corresponding cumulative attack rate (right) when 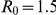 in the first wave with school closure. Lines correspond to no treatment (black) or treatment following profile (i–v) (blue, red, green, pink, yellow) in the first wave when 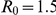. (a) No drug or vaccine. (b) With drug but no vaccine. Drug uptake is 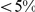. (c) With drug but no vaccine. Drug uptake is 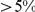.

Second wave without vaccination - Fig. 11 shows the level of infection (left) and cumulative attack rate (right) over the first 180 days of the second wave when vaccination is not used as a control strategy in the second wave. This figure demonstrates that when no treatment is used in the first wave (black line) this results in the lowest number of infections in the second wave by a considerable margin. This is true when treatment is not used in the second wave (Fig. 11(a)), treatment levels are low (Fig. 11(b)) and when treatment levels are high (Fig. 11(c)). Thus, when drug therapy is used in the first wave, the severity of the second wave is greatly increased. This is similar to the situation observed without school closure. Note that school closure makes profile (ii) and profile (v) have almost the same effect on the attack rate whether there is use of drug or not in the second wave.

**Figure 12:**
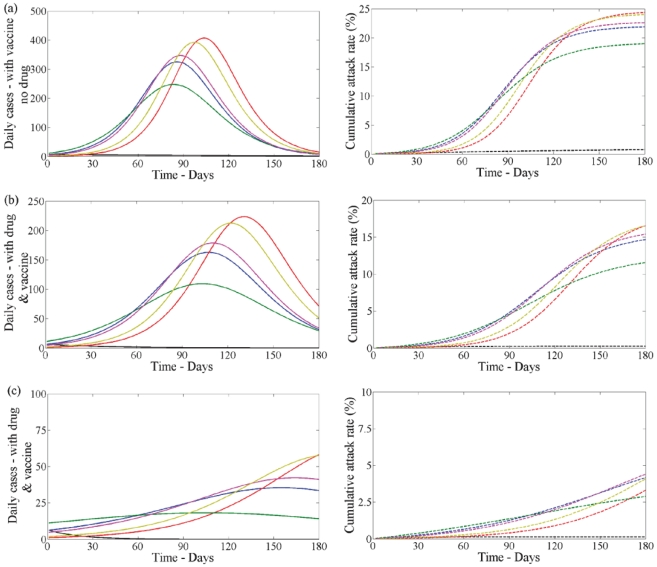
Disease incidence in second wave with vaccination and with school closure in first wave. Clinical infection in second wave with vaccine (left) and corresponding cumulative attack rate (right) when 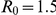 in the first wave with school closure. Lines correspond to no treatment (black) or treatment following profile (i–v) (blue, red, green, pink, yellow) in the first wave when 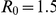. (a) With vaccine but no drug. (b) With drug and vaccine. Drug uptake is 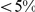. (c) With drug and vaccine. Drug uptake is 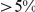.

Second wave with vaccination - Vaccination gives the same qualitative features as it did when school closure was not considered. When only vaccination is used the peak of the second wave is reduced in magnitude and occurs later in time compared to the case when only drug therapy is used (Fig. 11(a) and Fig. 12(a)). In turn, it reduces the cumulative attack rate in the second wave (Fig. 12(a) (right). When drug therapy and vaccine are both used in the second wave the total level of infection and cumulative attack rate are also reduced when compared to the case when vaccination was not used in the second wave (Fig. 11(b,c) and Fig. 12(b,c)).

The second wave is almost non-existent when drug therapy is not used in the first wave (Fig. 12(a,b,c)). If drug therapy is used in the first wave profile (iii) results in the lowest level of infection in the second wave(Fig. 12(a,b,c) green line). This is also true when school closure is not considered (Fig. 6, green line).

#### Both waves

**Figure 13:**
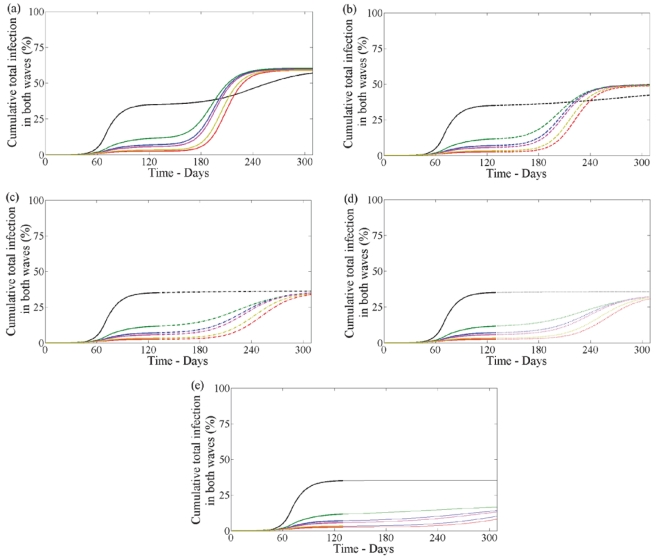
Cumulative total infections in both waves with school closure in first wave. Cumulative infections (clinical and subclinical) in both waves when 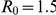 in the first wave with school closure. The five panels correspond to second wave: (a) no drug or vaccine (solid line), (b) no vaccine with drug 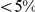 (dashed line), (c) no vaccine with drug 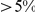 (dashdot line), (d) with drug uptake 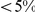 and vaccine coverage 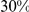 (dotted line), (e) with drug uptake 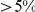 and vaccine coverage 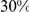 (solid line with circles). In each panel lines correspond to no treatment (black) and treatment profile (i–v) (blue, red, green, pink, yellow) in the first wave.

The cumulative number of infections (asymptomatic, symptomatic untreated and symptomatic treated on either day) over both waves for all treatment profiles in the first wave, and all combinations of drug therapy and vaccination in the second wave is shown in Fig. 13. This figure shows that the reduced transmissibility due to school closure over the summer months during the first wave reduces the total cumulative number over both waves when compared to the case when school closure did not occur (Fig. 9 and Fig. 13). The total cumulative infections when school closure occurs lies between 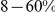 of the population depending on the control strategies used in both waves.

Comparing all combinations of strategies in the first and second waves, we find that a control strategy of vaccination and a high use of drug therapy in the second wave will always result in the lowest number of cases no matter what treatment profile is used in the first wave (Table 5(a)). This is also the case when school closure was not considered. However, the lowest number of total infections when considering school closure results from a combination of treatment profile (ii) in the first wave and vaccination and 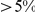 drug uptake in the second wave. This differs from our previous result where treatment profile (iii) in the first wave and vaccination and 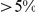 drug uptake in the second wave resulted in the lowest number of cases. Note that in all scenarios profile (iii) results in a higher number of infections than profile either or both of profile (iv) or (v). Thus, using treatment profile (iii) should not be considered if school closure over the summer months coincides with the first wave of infection.

**Table 5:** Cumulative (a) total infections and (b) clinical cases over both waves for 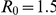 in first wave when schools are closed at the day 71 for 60 days (which marks the end of the first wave).

			Second wave				
			no drug	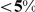	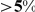	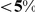	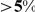
			no vaccine	no vaccine	no vaccine	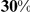	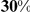
(a)	First wave	no treatment	57.0	42.3	36.2	35.6	35.3
		profile (i)	59.8	49.4	34.9	32.2	14.2
		profile (ii)	58.9	48.9	33.8	30.7	8.1
		profile (iii)	60.3	49.5	34.6	31.7	16.7
		profile (iv)	59.5	49.2	34.8	32.1	13.2
		profile (v)	59.1	48.9	34.4	31.5	10.2
(b)	First wave	no treatment	33.3	25.1	21.7	21.3	21.1
		profile (i)	35.3	29.3	20.7	18.8	8.4
		profile (ii)	35.2	29.3	20.2	18.0	4.8
		profile (iii)	35.6	29.2	20.5	18.5	9.9
		profile (iv)	35.4	29.3	20.7	18.8	7.8
		profile (v)	35.3	29.2	20.5	18.5	6.0

When vaccination is used in the second wave, or when vaccination is not used and drug therapy is high in the second wave, profile (ii) results in the lowest level of total infection (Table 5(a)). However, when vaccination is not used in the second wave and drug therapy use during the second wave is low or nonexistent then no treatment in the first wave results in the lowest cumulative number of infections over both waves (Table 5(a)).

Table 5(b) gives the cumulative number of clinical cases (treated and untreated) over both waves. The results here are the same as in that found for (Table 5(a)).

## Discussion

Pandemic preparedness is a public health priority and, with the recent emergence of the highly pathogenic H1N1 influenza virus, it has become even more important to define control policies that will effectively stave off infection of a population, or at least, greatly reduce the number of infections and the burden on the health care system. The control policy that a particular country may adapt will depend greatly on the resources of that country and, perhaps, on the generousity of other countries. Thus, the control policy that is adapted will differ between countries with high or low levels of antiviral stockpiles, and high or low levels of vaccine doses being purchased. The efficacy of the control policy will depend on the rates of uptake of treatment or prophylactic use of these antivirals, the rate of uptake of antiviral drugs (presentation to a doctor), the distribution of the vaccine and vaccine uptake. The efficacy of a control policy will also depend on the circulating strain, especially in successive waves since individuals infected in previous waves will have acquired some immunity.

A number of epidemiological models have explored various mitigation strategies for pandemic influenza throughout the globe. However, with the exception of [9] these have focussed on a single wave of infection. We developed a model that describes the first and second wave of an influenza pandemic which includes the two major interventions that can be taken during a pandemic, antivirals and vaccination. The model was used to assess the impact of different combinations of these on the severity of the first and second waves, and on the total number of infections over both waves for two scenarios when the first wave coincides with school closure over the summer months and when it does not. The first wave model includes the use of antiviral, where antivirals are not used, or one of five different treatment profiles is used. Each scenario was chosen to reflect possible use of countries with no antiviral stockpile (so no use of antivirals), a small stockpile (profile (iii)), a medium sized one (profile (i)), or a very large one (profile (ii)). We also explored the effects of changing treatment profile (i) to similar profiles of (ii) and (iii) where the total probability of treatment over the WOP was the same (treatment profiles (iv–v)). The outcome of the first wave is a susceptible population that has varying degrees of immunity gained from infection. This population is used as the initial population of the second wave of infection. In the second wave model we explored different combinations of drug treatment rates and vaccination uptake on the level of infection, including the effects of prior immunity from the first wave. This was done for two different values of of transmission 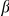, when school closure was not considered so as to capture the possibility of more fit influenza strains in the second wave.

We find that no matter what treatment strategy is used in the first wave, a combination of vaccination and 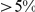 drug uptake in the second wave will result in the lowest amount of infection and clinical cases. This is lowest when treatment profile (iii) is used in the first wave when school closure is not considered and it is lowest when treatment profile (ii) is used in the first wave when summer school closure is included in the model.

In cases where vaccination and drug therapy are not readily available the optimal treatment strategy changes. When school closure is not considered the model predicts that if drug uptake is low in the second wave and vaccination is available, the total number of infections and clinical cases will be reduced if no drug therapy is used in the first wave. This result is also found in cases where drug therapy is available in the second wave but vaccination is not. However, if neither drug therapy or vaccination is available in the second wave, then treatment profile (ii) in the first wave will result in the lowest number of infections and clinical cases. The results vary when schools are closed over the summer months. Here, the model predicts that profile (ii) in the first wave will result in the lowest number of infections in all cases except when vaccination is not availble in the second wave and drug therapy use is low or non-existent.

Though the cost-effectiveness analysis of the proposed mitigation strategies has not been explicitly included in the present model, it is assumed that this is related to the total number of infections and clinical cases (Fig. 9, 13 and Table 4, 5). The economic evaluation of inter-pandemic influenza programs is an important issue in aspect of pandemic preparedness and our model could be compared with the economic evaluation of mitigation strategies from a social perspective in the USA [39] and Europe [9].

Though the parameter values introduced to reflect a reduction in susceptibility, a reduction in infectiousness and the probability of symptomatic infection in the second wave model are to some extent based on assumptions underlying the model, we conceive that the model results are significant and will aid in future directions in policy making for pandemic preparedness. In this context, however, more detailed model validation and parameter estimation using data from the current H1N1 pandemic or past pandemics should be a priority for future work. In the context of pre-existing immunity our results could be reviewed with some other modelling of mitigation strategies where it was predicted that pre-existing immunity in 15% or more of the population kept the attack rates low even if the whole population was not vaccinated or vaccination was delayed [22].

Immuno-epidemiology is an emerging field which studies the effects of individual immunity on disease dynamics at the population level. Recently, Heffernan and Keeling [25], [40] used a model describing the pathogenesis of measles in-host to parameterize an epidemiological model of measles infection to study the effects of vaccination and waning immunity on disease prevalence and asymptomatic infections. A similar study for influenza would be helpful in determining the level of immunity gained after influenza infection, studying how partial immunity aids in the protection of an individual against future strains of influenza, and studying how different distributions of pre-existing immunity from previous infection or vaccination in a population may provide herd immunity.

Future extensions of our model can include the study of the effects of other non-pharmaceutical intervention strategies on the severity of the first and second waves of a pandemic. These can include other scenarios of school closure that were not considered here, case isolation, household quarantine and restrictions on travel. However, the inclusion of one or more of these intervention strategies will add greatly to the complexity of the model.

The evolution of drug resistance is not explicitly considered in our study, but with the consideration of a higher 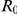 in the second wave we do account for the better strain fitness that is able to overcome some of the immunity gained from previous infection or vaccination. Previous modeling studies on the effects of the accumulation of drug resistant mutations have, like our study, found that use of drug therapies should be minimized so as to prevent large epidemics where drug therapy has no effect [24], [41]–[43].

In conclusion, the two wave model predicted that if drug therapy is readily available and a vaccine is available in the second wave, then profile (iii) or (ii) combined with this will result in the lowest number of possible infections in the population. However, the use of no treatment in the first wave is optimal in most cases when the drug therapy stockpile is limited, drug therapy use is low and if vaccination is not available. These results pertain to the population setting. The best result for an individual in the population is to prevent or stave off severe infection. In future work the benefits of the individual against the population will be weighed. This may affect the level of drug therapy use predicted by the model.

## Supporting Information

Text S1 Influenza pandemic waves under various mitigation strategies with 2009 H1N1 as a case study.
(0.04 MB PDF)
