# Supplementary material for: Influenza Pandemic Waves under Various Mitigation Strategies with 2009 H1N1 as a Case Study
Source: PLoS One. 2010 Dec 20;5(12):e14307. doi: 10.1371/journal.pone.0014307 (PMC3004963; doi:10.1371/journal.pone.0014307)
Supplement: Text S1 — Influenza pandemic waves under various mitigation strategies with 2009 H1N1 as a case study. (0.04 MB PDF) [file pone.0014307.s001.pdf]

Supplementary Information : Influenza Pandemic waves under various mitigation strategies with 2009 H1N1 as a case study

Suma Ghosh, Jane Heffernan,

This supplementary material provides the derivation of the infected classes in the first wave model and the force of infection with treatment in WOP which is divided into two stages: early and late WOP.

1. Derivation of two infected classes in the first wave model

Let a be the time elapsed since the onset of symptoms and $i_{U_j}(t,a), i_{T_j}(t,a)$ be the densities of untreated and treated individuals respectively in j-th phase ($j = 1, 2$) of infection age a at current time t. Presuming the treatment rate $r_j(a)$ of respective phases at infection age a after the onset of symptoms, we have for the early stage i.e., for $a \in [0, \frac{n}{2}]$ (n being length of wop)

$$\left(\frac{\partial}{\partial t} + \frac{\partial}{\partial a}\right) i_{U_1}(t,a) = -r_1(a) i_{U_1}(t,a) \tag{1}$$

$$\left(\frac{\partial}{\partial t} + \frac{\partial}{\partial a}\right) i_{T_1}(t,a) = r_1(a) i_{U_1}(t,a) \tag{2}$$

subject to the boundary conditions

$$i_{U_1}(t,0) = p\mu_E E(t), \quad i_{T_1}(t,0) = 0 \tag{3}$$

and for the second phase or late wop, i.e., for $a \in [\frac{n}{2}, n]$

$$\left(\frac{\partial}{\partial t} + \frac{\partial}{\partial a}\right) i_{U_2}(t,a) = -r_2(a) i_{U_2}(t,a) \tag{4}$$

$$\left(\frac{\partial}{\partial t} + \frac{\partial}{\partial a}\right) i_{T_2}(t,a) = r_2(a) i_{U_2}(t,a) \tag{5}$$

with the following boundary conditions

$$i_{U_2}(t,0) = i_{U_1}(t, \frac{n}{2}), \quad i_{T_2}(t,0) = 0 \tag{6}$$

Solving equations (1), (2) and using the boundary condition (3) we get for $t \geq a$,

$$i_{U_1}(t,a) = p\mu_E E(t-a) q(a) \tag{7}$$

$$i_{T_1}(t,a) = p\mu_E E(t-a)(1-q(a)) \tag{8}$$

Also solving (4), (5) subject to (6) we get for $t - \frac{n}{2} \geq a$,

$$i_{U_2}(t,a) = p\mu_E q(\frac{n}{2}) E(t - \frac{n}{2} - a) q'(a) \tag{9}$$

$$i_{T_2}(t,a) = p\mu_E q(\frac{n}{2}) E(t - \frac{n}{2} - a)(1 - q'(a)) \tag{10}$$

where

$$q(a) = e^{-\int_0^a r_1(x)dx}, \quad 0 \leq a \leq \frac{n}{2} \tag{11}$$

$$q'(a) = e^{-\int_{\frac{n}{2}}^{a+\frac{n}{2}} r_2(x)dx}, \quad 0 \leq a \leq \frac{n}{2} \tag{12}$$

Now let $I_{U_1}(t)$ and $I_{T_1}(t)$ denote the total number of untreated and treated individuals respectively after the first phase of wop, then for $t \geq n/2$ we have

$$\begin{aligned}
\frac{dI_{U_1}(t)}{dt} &= i_{U_1}(t, \frac{n}{2}) - (\mu_{U_1} + d_{U_1})I_{U_1} && (13)\\
&= p\mu_E E(t - \frac{n}{2})q(\frac{n}{2}) - (\mu_{U_1} + d_{U_1})I_{U_1} && (14)\\
\frac{dI_{T_1}(t)}{dt} &= i_{T_1}(t, \frac{n}{2}) - (\mu_{T_1} + d_{T_1})I_{T_1} && (15)\\
&= p\mu_E E(t - \frac{n}{2})(1 - q(\frac{n}{2})) - (\mu_{T_1} + d_{T_1})I_{T_1} && (16)
\end{aligned}$$

Similarly the total number of untreated and treated individuals after the second phase of wop, i.e., for $t \geq n$ are given by

$$\begin{aligned}
\frac{dI_{U_2}(t)}{dt} &= i_{U_2}(t, \frac{n}{2}) - (\mu_{U_2} + d_{U_2})I_{U_2} && (17)\\
&= p\mu_E q(\frac{n}{2})E(t - n)q'(\frac{n}{2}) - (\mu_{U_2} + d_{U_2})I_{U_2} && (18)\\
\frac{dI_{T_2}(t)}{dt} &= i_{T_2}(t, \frac{n}{2}) - (\mu_{T_2} + d_{T_2})I_{T_2} && (19)\\
&= p\mu_E q(\frac{n}{2})E(t - n)(1 - q'(\frac{n}{2})) - (\mu_{T_2} + d_{T_2})I_{T_2} && (20)
\end{aligned}$$

2. Force of infection in the first wave model

As we assume that antiviral treatment starts onset of infection and continues during the period of wop, the treated individuals transmit the infection with reduced force of infection. So, the resultant force of infection due to all compartments during the period can explicitly be expressed as

$$\begin{aligned}
\Lambda(t) &= \delta_A A(t) + \delta_U I_U(t) + \delta_U \delta_{T_1} I_{T_1} + \delta_U \delta_{T_2} I_{T_2} + \int_0^{\frac{n}{2}} [i_{U_1}(t, a) \\
&+ \delta_{T_1} i_{T_1}(t, a)]da + \int_{\frac{n}{2}}^{n} [i_{U_2}(t, a) + \delta_{T_2} i_{T_2}(t, a)]da, \quad t \geq 0 && (21)
\end{aligned}$$

where δ_A, δ_U are the relative transmissibility of asymptomatic and symptomatic but untreated, δ_{T_1}, δ_{T_2} are the reduction in infectiousness due to treatment on first and second phase of wop respectively. $I_U(t) = I_{U_2}(t)$ represent the total infected individuals remain untreated at last i.e., at the end of wop and $\mu_U = \mu_{U_1} = \mu_{U_2}$, $d_U = d_{U_1} = d_{U_2}$.

In absence of treatment and considering the initial exposed individual $E(0)$ to be very small compared to the initial susceptible population $S(0)$ the final size relation [1] can be approximated to

$$log_e(S_0/S_\infty) = R_0(1 - S_\infty/S_0) \quad (22)$$

This relation can be used to estimate R_0 based on the clinical attack rate - the fraction of the susceptible population that develops disease symptoms during the course of epidemic - given by the expression $p(1 - S_\infty/S_0)$ where S_∞ is the size of the susceptible population when the epidemic dies out.

References

[1] Arino J, Brauer F, van den Driessche P, Watmough J, Wu J (2007) A final size relation for epidemic models. Math Biosc and Eng 4 (2): 159-175.
